# Rare Pediatric Posterior Stroke Case Report with Discussion of Brainstem Lesions

**DOI:** 10.3390/neurolint17110178

**Published:** 2025-11-03

**Authors:** Lauren A. Gould, Matthew Carman, Gian Rossi, Jasvinder Dhillon

**Affiliations:** 1 Emergency Medicine, Lakeland Regional Hospital, 1324 Lakeland Hills Blvd, Lakeland, FL 33805, USA; 2 Pediatric Neurology, Nemours Children’s Hospital, Orlando, FL 32827, USA; 3 Pediatric Critical Care, Lakeland Regional Hospital, Lakeland, FL 33805, USA

**Keywords:** pediatric stroke, posterior circulation stroke, brainstem stroke, pontine stroke, patent foramen ovale (PFO), risk factors for pediatric stroke, ischemic stroke, cryptogenic stroke, Brainstem Rules of Four

## Abstract

**Introduction:** The rates of pediatric ischemic stroke incidence have more than doubled over the past 3–4 decades; however, pediatric posterior circulation strokes are even more uncommon. These rising incidence rates have led to increasing awareness of pediatric strokes and the development of institutional guidelines regarding these patients to optimize outcomes when possible. **Case Report:** We describe a rare case of acute ischemic posterior circulation stroke in a 14-year-old previously healthy adolescent boy who presented with right-sided facial droop, dysarthria, and right-sided hemiplegia. An MRI of the brain demonstrated an acute infarct in the brainstem, and an echocardiogram demonstrated a patent foramen ovale (PFO). We also discuss how to localize brainstem lesions to a specific location within the brainstem and associated blood supply using symptomatology. **Conclusions:** All stroke patients require evaluation for possible etiologies of stroke and possible underlying risk factors. Nearly half of patients who suffer from cryptogenic stroke are found to have a PFO, and adult studies have shown that PFO closure is associated with reduced recurrent cryptogenic strokes, although pediatric-specific data is lacking. If a posterior stroke is suspected, specifically in the brainstem, then the Brainstem Rules of Four may be utilized to localize these lesions and identify blood supply using simplified knowledge of the brainstem anatomy.

## 1. Background

Pediatric strokes are a cause of significant morbidity and mortality amongst pediatric patients. The rates of pediatric ischemic stroke incidence have more than doubled over the past 3–4 decades, with an annual incidence rate today of approximately 4.6–6.4 per 100,000 children [1,2,3,4,5,6]. Posterior circulation ischemic strokes in the cerebellum, brainstem, and posterior cerebral cortex are extremely rare in children, accounting for 15–22% of all pediatric ischemic strokes, with an incidence rate of approximately 0.2–0.38 per 100,000 children [7,8,9]. The time from symptom onset to diagnosis of stroke in pediatric patients can be delayed by a lack of parental recognition of often subtle neurological symptoms, clinicians not considering stroke as a differential diagnosis, a desire to avoid radiation in children, or a lack of emergent MRI scans [10]. We describe a case of acute ischemic stroke in a 14-year-old previously healthy adolescent boy who presented with dysarthria, right-sided facial droop, and right hemiplegia. An MRI demonstrated a stroke within the brainstem, and an echocardiogram demonstrated a patent foramen ovale (PFO). We also discuss how to localize brainstem lesions to a specific location within the brainstem and associated blood supply within each area using symptomatology.

## 2. Case

The patient was a previously healthy 14-year-old right-handed boy, who arrived via EMS with a potential pediatric stroke alert due to right-sided facial droop, dysarthria, and right-sided flaccidity of his arm and leg. The patient woke up around 11 a.m. on the day of presentation with right-sided body weakness, and he was unable to move or get out of bed. He was able to reach his phone, and called his parents, who subsequently called 911. According to the patient and his parents, his last known normal was the night prior to arrival at 10 p.m. At that time, he noted that the right side of his body began to feel weaker and slightly numb. The patient ignored his symptoms and went to bed, but when he woke up his symptoms had significantly worsened. The patient also reflected that he had a field day outside at school on the day prior to presentation where he was “dizzy” throughout the day, and he fell down during activities several times which was unusual for him. His dizziness and decreased coordination with associated falls were likely early posterior fossa stroke symptoms. Additional family history included only cardiovascular disease on the patient’s maternal side.

On physical exam, the patient had slurred speech and right-sided facial droop. Motor strength was normal on the left side of his body but was 0/5 in his right proximal upper extremity and 0/5 in the distal right upper extremity. The right lower extremity had 0/5 strength. He complained of tingling and mild numbness in the right upper and lower extremities and right lower face.

**Figure 1:**
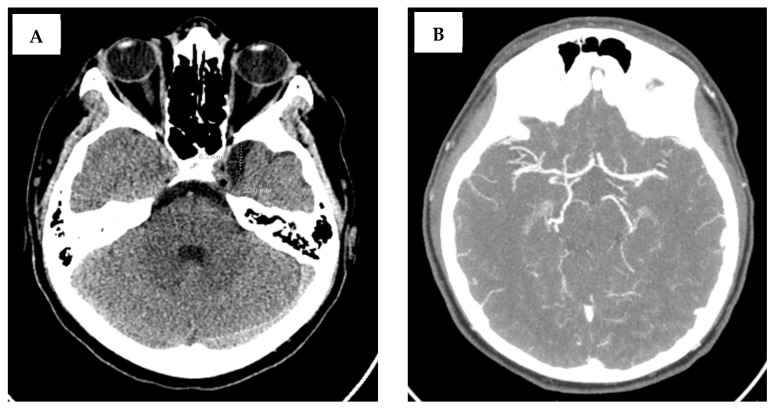
Patient CT imaging of the brain: CT without contrast (**A**) demonstrated a small arachnoid cyst in the left middle cranial fossa measuring 2.2 × 0.8 cm highlighted by dotted lines within figure. There were no acute infarcts, masses, or hemorrhages. CT angiogram of the brain (**B**) was unremarkable, with no occlusion, stenosis, or aneurysm of the cerebral vasculature.

Pediatric neurology was consulted, and a non-contrast head CT and CT angiogram was performed. Non-contrast head CT demonstrated an incidental small arachnoid cyst but was otherwise unremarkable, as shown in Figure 1. The CT angiogram of the head and neck was unremarkable, without any evidence of stenosis, occlusion, or aneurysm (Figure 1). The patient was not given thrombolytics due to being outside of the treatment time window, although pediatric thrombolytic therapy is included in our Institutional Pediatric Stroke Pathway. Our Institutional Pediatric Stroke Pathway includes thrombolytics within 4.5 h and thrombectomy for large-vessel occlusions within 24 h. While our patient presented within 24 h of symptom onset, revascularization with endovascular intervention was not possible due to the distal location within the brainstem.

**Figure 2:**
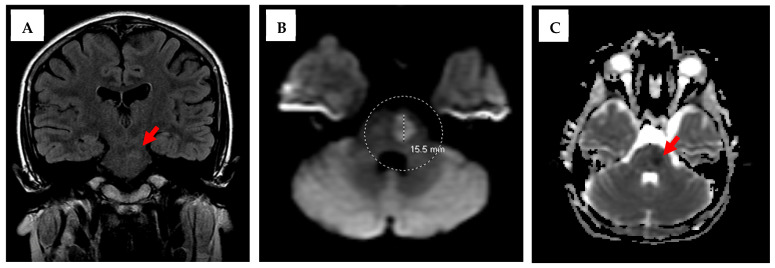
Patient MR imaging of the brain: Coronal view of the T2 FLAIR MRI on the left (**A**) demonstrating a hyperintense subacute infarct in the left pons (red arrow). Axial view of the diffusion-weighted MRI in the middle (**B**) circle and dotted line further demonstrating a 1.5 × 1.0 cm acute/subacute infarct in the left paramedian pons. Axial view of an apparent diffusion coefficient (ADC) map of the MRI to the right (**C**) with a diminished ADC signal (red arrow) corresponding with the subacute infarct.

The chest X-ray was also unremarkable. Labs, including a complete blood count (CBC), a comprehensive metabolic panel (CMP), coagulation studies, and urine drug screening, were all unremarkable. The patient was subsequently admitted to the pediatric intensive care unit (PICU) for neurologic checks every hour, close monitoring, and further evaluation with a brain MRI. The brain MRI demonstrated a 1.5 × 1.0 cm acute/subacute ischemic infarct in the left paramedian pons, as seen in Figure 2.

On day 2 in the PICU, the patient’s motor strength was improving though not back to baseline. The patient still had slurred speech, but it was significantly improved from the previous day. Right-sided facial droop persisted. The proximal strength of his RUE was 3/5, and that of his distal RUE was still 0/5. The motor strength of his right hip was 3/5, and the distal RLE was 4/5.

The patient underwent an echocardiogram that demonstrated a patent foreman ovale. It was decided that the patient would be transferred to a tertiary pediatric center for further evaluation and management. After a negative hypercoagulability workup and normal Doppler ultrasounds of the upper and lower extremities, the patient was ultimately diagnosed with an embolic stroke of unknown source and transferred to an inpatient rehabilitation center for continued Physical and Occupational Therapy.

## 3. Discussion

This case involved a 14-year-old boy who presented with significant neurologic deficits after suffering from an acute left paramedian pontine stroke within the brainstem. Our patient had a delay in diagnosis due to a postponement in seeking treatment, although he demonstrated early signs of stroke with his episodes of “dizziness” and falls while at school, which were likely early posterior fossa symptoms. Our patient was found to have a patent foramen ovale (PFO) on echocardiogram, which can provide a portal for a thrombus to pass from the right side of the heart to the left and travel to the brain, causing a stroke. PFO is associated with cryptogenic stroke (stroke of unclear etiology) and results most commonly from a paradoxical embolism in which an embolus is carried from the venous circulation to the arterial circulation [11,12]. In all patients who suffer from cryptogenic strokes, 40–50% of them have a PFO, and adult studies have shown that PFO closure can significantly reduce the risk of recurrent stroke, although pediatric data is lacking [12,13,14]. There have been multiple case reports of pediatric patients with PFOs diagnosed with cryptogenic stroke; however, guidelines for pediatric patients are lacking [15]. The Risk of Paradoxical Embolism (RoPE) score has been validated as a tool to identify stroke-related PFO in adult patients with cryptogenic stroke, and patients receive a higher score for younger age indicating that younger patients with PFOs and diagnosed with cryptogenic stroke are associated with a higher chance of their stroke being related to the PFO; although this has not been extrapolated to the pediatric population, it may be useful [16].

The differential diagnosis for stroke includes ischemic stroke, hemorrhagic stroke, transient ischemic attack (TIA), seizure (sometimes associated with a postictal Todd’s paralysis), hypoglycemia, brain tumor/lesion, migraine with aura, meningitis/encephalitis, acute demyelinating syndromes, conversion disorder, vestibular neuritis, toxic encephalopathies, and peripheral neuropathies. The risk factors for stroke in pediatric patients vastly differ from those in adults. Adult risk factors include tobacco smoking, hypertension, hyperlipidemia, diabetes mellitus, and obesity. Stroke risk factors for children include arteriopathies, cardiac problems, hematologic/hypercoagulable states, genetic/metabolic disorders, infections, and others, as further described in Table 1. Aside from the PFO, our patient’s additional identified possible risk factors throughout his workup only included being overweight, weighing 94 kg with a body mass index of 30 kg/m^2^.

Our Institutional Pediatric Stroke Pathway includes thrombolytics within 4.5 h and thrombectomy for large-vessel occlusions within 24 h; however, revascularization with endovascular intervention was not possible for our patient, although his symptoms began within 24 h due to the distal location within the brainstem. Our patient did not require neurosurgical intervention, although it should be highlighted that there is high risk of brainstem compression from these lesions due to post-stroke vasogenic edema that occurs in the days following the initial insult. Patients require close monitoring for worsening symptoms, as a decompressive craniectomy may be required if edema progresses to brainstem compression.

Our patient presented with a posterior circulation arterial ischemic stroke of the left paramedian pons that resulted in right-sided lower facial droop and hemiplegia and dysarthria with normal extraocular movements. Descending corticospinal and corticobulbar tract involvement explained the symptoms, with sparing of the tegmentum and cranial nerve nuclei and tracts. Brainstem lesions can be localized using patient symptomatology and then by localizing the lesion, and the most commonly affected blood supply can also be identified. A method for localizing brainstem lesions known as the “Brainstem Rules of Four” was developed by Dr. Peter Gates, an Australian neurologist, in 2005 to simplify our understanding of the brainstem anatomy. The four rules are listed below and summarized in further detail in Table 2 with associated deficits when lesions are present [17].

*Rule #1*: There are four structures in the “midline” beginning with **M,** and they are mostly responsible for **m**otor function.

*Rule #2*: There are four structures to the “side (lateral)” beginning with **S,** and they mostly responsible for **s**ensory function.

*Rule #3*: There are four cranial nerves (CNs) in the medulla, four in the pons, and four above the pons (two in the midbrain and two in the cerebrum).

*Rule #4*: The four motor nuclei that are in the midline are those that divide equally into 12 (except CNs 1 and 2 located within the cerebrum). So, CNs 3, 4, 6, and 12 are located in the midline. Other cranial nerves, including CNs 5, 7, 8, 9, 10, and 11, are located laterally.

Please refer to Table 2 for a concise summary of the Brainstem Rules of Four that can be utilized to localize brainstem lesions and includes associated blood supplies for each location. A caveat should be noted that CN 5 (trigeminal nerve) and CN 8 (vestibulocochlear nerve) both run beyond the pons and should therefore not be used to localize lesions to the pons area. Instead, loss of ipsilateral pain/temperature sensation to the face (CN 5) or hearing loss (CN 8) should only be used to localize the lesions laterally.

**Figure 3:**
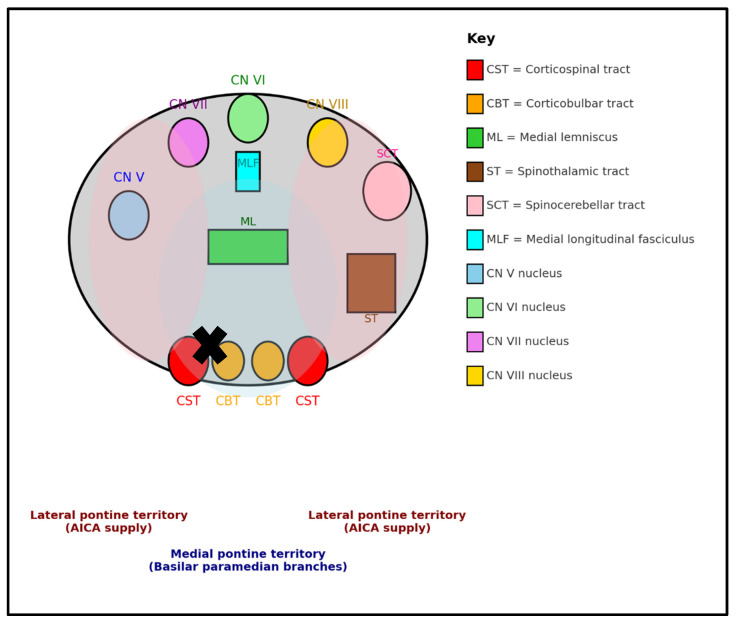
Schematic of a cross-section of the pons within the brainstem with tracts, nuclei, and vascular zones. **X** indicates the location of our patient’s lesion. Medial blood supply is from branches of the basilar artery and lateral from the anterior inferior cerebellar artery (AICA).

When applying the Brainstem Rules of Four to our patient, his deficits were primarily motor-based, indicating a medial lesion involving the corticospinal tract. His symptoms could further be localized to the pons due to his facial droop, indicating involvement of cranial nerve 7 (facial nerve), although it was likely the corticobulbar tract extension, not the cranial nerve nucleus itself, that was affected. Cranial nerve 7, according to the rules, is considered to be located laterally; however, our patient’s lesion was located “paramedian”, creating a mixed picture. This is further visualized in Figure 3, which includes a schematic cross-section of the pons with tracts, nuclei, and vascular zones.

## 4. Conclusions

The rates of pediatric ischemic stroke incidence have more than doubled over the past 3–4 decades; however, they remain rare, and posterior strokes in pediatric patients are extremely uncommon. Consideration of stroke in diagnostic differentials when pediatric patients present with headaches, dizziness, or focal neurologic symptoms is paramount, and if suspected, appropriate imaging should be ordered regardless of radiation risks. Development of institutional guidelines for possible pediatric strokes may optimize outcomes. All stroke patients should have an echocardiogram completed to evaluate for anatomic abnormalities and possible etiologies of stroke, and an evaluation of possible risk factors should be performed, including underlying hypercoagulable states. If a PFO is identified, then closure may be considered, as it is associated with a reduced risk of recurrent cryptogenic stroke. Patients suffering from posterior circulation strokes require close monitoring for worsening symptoms due to risk of post-stroke cerebral edema in the following days, which may lead to brainstem compression requiring neurosurgical intervention for decompression. If a posterior stroke is suspected, specifically in the brainstem, the Brainstem Rules of Four may be utilized to localize these lesions and identify blood supply using simplified knowledge of the brainstem anatomy.

## Figures and Tables

**Table 1:** Risk factors for childhood ischemic stroke.

Types	Examples
Arteriopathies	- Moyamoya disease - Craniocerebral artery dissection (internal carotid or vertebral artery dissection) - Arteriovenous malformation (AVM) - Focal cerebral arteriopathy of childhood (FCA) - Fibromuscular dysplasia
Cardiac Problems	- Congenital heart disease - Valvular heart disease - Cardiomyopathy - Dysrhythmia - Catheterization or cardiac surgery - Hypertension
Hematologic/Hypercoagulable states/Thrombophilia	- Sickle cell disease - Protein C, protein S, and antithrombin deficiency - Factor V Leiden - Prothrombin G20210A - MTHFR C677T - Lipoprotein A elevation - Antiphospholipid syndrome - Systemic vasculitis
Genetic/Metabolic Disorders	- Connective tissue disorders (i.e., Marfan, Ehlers Danlos) - Trisomy 21 - Neurofibromatosis type 1
Infection	- Meningitis - Encephalitis - Varicella (up to 1 year post-infection)
Neonates (Perinatal stroke, 0–28 days)	Note: Neonates can present with seizure or seizure-like symptoms, and 10% of term neonatal seizures are secondary to perinatal strokes.
Other	- Toxicology (cocaine, amphetamines) - Medications (birth control) - Immobility - Obesity - Severe dehydration - Head or neck trauma

**Table 2:** Brainstem Rules of Four summary with associated blood supplies [17].

Brainstem	Localization	Blood Supply	Cranial Nerves (CN)	Symptomology from CN Lesion	Structures & Symptomology from Structure Lesion (Applies to Entire Brainstem, Not Specific to One Area)
**Midbrain**	Medial	Posterior cerebral artery (PCA)	CN 3 (oculomotor) CN 4 (trochlear)	- Eye turned out and down - Eye unable to look down when looking towards nose	Medial Structures (primarily motor): - Corticospinal tract (motor pathway) → Contralateral weakness - Medial lemniscus → Contralateral loss of proprioception/vibration - Medial longitudinal fasciculus → Ipsilateral internuclear ophthalmoplegia (INO) - Motor nucleus and nerve → Ipsilateral CN motor loss (CN 3, 4, 6, & 12)
	Side (Lateral)	PCA	None	-	
**Pons**	Medial	Anterior inferior cerebellar artery (AICA)	CN 6 (abducens)	- Ipsilateral eye abduction weakness	
	Side (Lateral)	Basilar artery	CN 5 (trigeminal) CN 7 (facial) CN 8 (vestibulocochlear)	- Ipsilateral facial sensory loss - Ipsilateral facial weakness/droop - Ipsilateral deafness	Side (Lateral) Structures (primarily sensory): - Spinocerebellar pathway → Ipsilateral ataxia - Spinothalamic tract → Contralateral loss of pain/temperature sensation - Sensory nucleus of CN5 → Ipsilateral loss of pain/temperature in the face - Sympathetic pathway → Ipsilateral Horner’s syndrome
**Medulla**	Medial	Posterior inferior cerebellar artery (PICA)	CN 12 (hypoglossal)	- Ipsilateral weakness of tongue	
	Side (Lateral)	Anterior spinal artery (ASA)	CN 9 (glossopharyngeal) CN 10 (vagus) CN 11 (accessory)	- Ipsilateral pharyngeal sensory loss - Ipsilateral palatal weakness - Ipsilateral shoulder weakness	

Note: The dark gray background represents primarily medial structures and the light gray background represents primarily lateral structures.

## Data Availability

Access to additional data regarding this case report can be made upon request to the authors. Some data may be restricted in order to protect patient privacy. Requests can be made to the corresponding author upon request.
